# Endometriosis and polycystic ovary syndrome are diametric disorders

**DOI:** 10.1111/eva.13244

**Published:** 2021-05-14

**Authors:** Natalie L. Dinsdale, Bernard J. Crespi

**Affiliations:** ^1^ Department of Biological Sciences Simon Fraser University Burnaby BC Canada

**Keywords:** anogenital distance, endometriosis, folliculogenesis, polycystic ovary syndrome, testosterone

## Abstract

Evolutionary and comparative approaches can yield novel insights into human adaptation and disease. Endometriosis and polycystic ovary syndrome (PCOS) each affect up to 10% of women and significantly reduce the health, fertility, and quality of life of those affected. PCOS and endometriosis have yet to be considered as related to one another, although both conditions involve alterations to prenatal testosterone levels and atypical functioning of the hypothalamic–pituitary–gonadal (HPG) axis. Here, we propose and evaluate the novel hypothesis that endometriosis and PCOS represent extreme and diametric (opposite) outcomes of variation in HPG axis development and activity, with endometriosis mediated in notable part by low prenatal and postnatal testosterone, while PCOS is mediated by high prenatal testosterone. This diametric disorder hypothesis predicts that, for characteristics shaped by the HPG axis, including hormonal profiles, reproductive physiology, life‐history traits, and body morphology, women with PCOS and women with endometriosis will manifest opposite phenotypes. To evaluate these predictions, we review and synthesize existing evidence from developmental biology, endocrinology, physiology, life history, and epidemiology. The hypothesis of diametric phenotypes between endometriosis and PCOS is strongly supported across these diverse fields of research. Furthermore, the contrasts between endometriosis and PCOS in humans parallel differences among nonhuman animals in effects of low versus high prenatal testosterone on female reproductive traits. These findings suggest that PCOS and endometriosis represent maladaptive extremes of both female life‐history variation and expression of sexually dimorphic female reproductive traits. The diametric disorder hypothesis for endometriosis and PCOS provides novel, unifying, proximate, and evolutionary explanations for endometriosis risk, synthesizes diverse lines of research concerning the two most common female reproductive disorders, and generates future avenues of research for improving the quality of life and health of women.

## 1 INTRODUCTION

Evolutionary processes shape human vulnerabilities to disease (Nesse & Stearns, 2008). Some pairs of diseases, such as autoimmunity and infection, cancer and neurodegeneration, and autism and psychosis, represent opposites to one another (Crespi & Go, 2015). Such diseases manifest in pairs because they involve dysfunction of the same general biological system, but in diametric directions. For example, higher risk of infection involves immune system under‐activity whereas higher risk of autoimmune disorder involves immune system overactivity (Crespi & Go, 2015). This framework of diametric diseases provides key insights into evolutionary and medical questions, as disease pairs and their symptoms can represent extreme, maladaptive expressions of adaptations and trade‐offs.

Endometriosis and polycystic ovary syndrome (PCOS) both involve altered functioning of the female hypothalamic–pituitary–gonadal (HPG) axis, but they have yet to be considered as associated with or related to one another. Here, we propose and evaluate the novel hypothesis that these two conditions represent a pair of diametric diseases. First, we describe what is known and unknown concerning the etiologies of endometriosis and PCOS. Second, we review how prenatal testosterone impacts HPG development and activity in females, specifying how different levels of prenatal testosterone should affect the HPG. Third, we describe in detail the diametric disorders hypothesis for endometriosis and PCOS risk. Fourth, we deduce six predictions from this hypothesis, reviewing each prediction through drawing on existing literature from multiple fields of study. Finally, we synthesize these diverse lines of evidence, evaluating the strength of support for the hypothesis, in addition to its implications and limitations.

The etiology of endometriosis, a condition diagnosed when endometrial tissue proliferates beyond the uterine boundaries, is highly enigmatic (Bulun et al., 2019; Burney & Giudice, 2012; Giudice & Kao, 2004). Women with endometriosis can experience severe pelvic pain, especially during menstruation (dysmenorrhea), and heavy menstrual bleeding, as well as reduced fertility (Bulun et al., 2019).

Research into the causes of endometriosis largely focuses on explanations for how endometrial cells appear in the peritoneal cavity and other bodily sites (Brosens et al., 2015; Sampson, 1927; Sasson & Taylor, 2008), identification of predisposing genes and environmental risk factors (García‐Peñarrubia et al., 2020; Rahmioglu et al., 2014), and studies of steroid and immunological pathways that promote lesion establishment and growth (García‐Gómez et al., 2020; Marquardt et al., 2019). These approaches help to explain aspects of the disorder, but a unifying explanation for why some women are particularly vulnerable to endometriosis remains elusive.

Relatively more is known concerning the etiology of PCOS than endometriosis. PCOS is diagnosed when women manifest signs of hyperandrogenism (high testosterone), irregular or absent ovulation, and polycystic (multi‐small follicle) ovaries; other traits, especially insulin resistance and obesity, frequently co‐occur (Rotterdam, 2004). These diverse symptoms of PCOS emerge following puberty, primarily in response to elevated androgenic activity of ovarian origin (Rosenfield & Ehrmann, 2016). In response to insulin, ovaries of women with PCOS produce especially high levels of androgens while peripheral tissues demonstrate insulin resistance. Resulting hyperinsulinemia further augments androgen production, contributing to obesity in women, which further increases androgen levels via adipose tissue (Diamanti‐Kandarakis & Dunaif, 2012). The metabolic and endocrine alterations observed in PCOS interact in complex ways and are rooted in early development: Fetal exposure to high levels of testosterone during an early, critical window of embryological development biases the HPG axis toward increased testosterone production that continues throughout postnatal life (Abbott et al., 2002, 2019; Filippou & Homburg, 2017; Walters et al., 2018).

The HPG axis regulates many aspects of reproduction in both sexes, including sex differentiation in utero, hormone production, and the pacing of reproductive events—including menarche and menopause in women—throughout postnatal life (Plant, 2015). Genitourinary structures, endocrine systems, and features of the nervous system develop in response to sexually dimorphic levels of testosterone, with male fetuses subject to substantially higher levels of testosterone than female fetuses.

In species bearing litters, females gestating beside males are commonly exposed to elevated levels of testosterone originating from the testes of their male littermates (Vandenbergh & Huggett, 1995). In species such as humans, which typically bear singleton offspring, variation in prenatal testosterone exposure of developing females comes from the fetus itself, the placenta, and the mother's circulation, such that testosterone accumulates from numerous sources including her adrenal glands, fat tissues, and ovaries (Hakim et al., 2017). Testosterone levels thus vary between individual females, in humans and other mammals, and have important, lifelong consequences on adult morphology, physiology, behavior, and reproduction via the development of the HPG axis (Bütikofer et al., 2019; Robinson, 2006; Ryan & Vandenbergh, 2002; Slutske et al., 2011; Tyndall et al., 2012).

Multiple causes contribute to female fetal exposure to testosterone. Maternally produced testosterone enters the placenta, but in typically developing females, most is converted into estrogens via aromatase (Kallak et al., 2017). The metabolic alterations of PCOS contribute to placental dysfunction in affected mothers, such that increased insulin secretion reduces placental aromatase activity, exposing female fetuses to elevated testosterone (Dumesic et al., 2020). Fetal ovaries also respond to maternal hyperinsulinemia, evident in women with diabetes or PCOS, by upregulating androgen production, representing a maternal–fetal interaction that increases overall testosterone exposure (Dumesic et al., 2020).

Other maternal factors such as elevated stress, quantified by amniotic cortisol levels, increased maternal weight gain, and young maternal age together predict 64.3% of the variation in amniotic testosterone drawn from female fetuses (Kallak et al., 2017). Further, evidence from twin studies of serum testosterone or hormonally mediated digit ratio measurements demonstrates that adult testosterone as well as its bioavailability demonstrate significant additive genetic effects, indicating that women who produce more testosterone will have offspring who also produce higher testosterone (Coviello et al., 2011; Hiraishi et al., 2012; Paul et al., 2006; Stone et al., 2009).

When developing female embryos are exposed to relatively high levels of testosterone—such as in females who develop PCOS—many aspects of HPG activity are affected. Hypothalamic sensitivity to steroid‐induced negative feedback is reduced, resulting in an increased frequency and amplitude of gonadotropin‐releasing hormone (GnRH) and luteinizing hormone (LH) pulses with corresponding increases in LH levels (Pastor et al., 1998; Roland & Moenter, 2014). Increased LH relative to follicle‐stimulating hormone (FSH) results in elevated ovarian testosterone with subsequent arrest of follicular maturation (Burt Solorzano et al., 2012). Immature follicles release high levels of anti‐Müllerian hormone (AMH), which further stimulate GnRH release while inhibiting FSH release (Franks & Hardy, 2020). The ovulation‐inducing LH surge is also impaired, resulting in lengthened or absent menstrual cycles (Foecking et al., 2005; Robinson, 2006). Prenatal androgen treatment induces HPG changes as well as metabolic alterations including insulin resistance, obesity, and enlarged adipocytes in female rhesus monkeys and mice (Abbott et al., 2009; Roland et al., 2010). Importantly, these testosterone‐induced alterations to female development are largely consistent across animal models (Astapova et al., 2019; Roland & Moenter, 2014).

Contributions of high prenatal testosterone to disease risks in women are thus well understood. Whether and how relatively *low* prenatal testosterone contributes to disease risk in women has yet to be explored in any detail, but several clues suggest that endometriosis may represent such an outcome. First, recent findings of short anogenital distances (AGDs) in women with endometriosis compared with controls (Crestani et al., 2020; Mendiola et al., 2016; Peters et al., 2020) implicate low prenatal testosterone in this disorder, although these results have yet to be considered or synthesized in the context of how and why endometriosis develops. Second, women with endometriosis demonstrate atypical HPG functioning (Cahill & Hull, 2000; Cahill et al., 1995; Stilley et al., 2012), which can reflect prenatal programming via hormonal exposure. Third, prenatal exposure to estrogenic and antiandrogenic chemicals are known to predict endometriosis risk (Missmer et al., 2004).

In contrast to the known HPG alterations induced by relatively high prenatal testosterone characteristic of PCOS, a disorder caused by relatively low prenatal testosterone should centrally involve the following alterations: (1) a lower frequency of GnRH pulses reflected by reduced LH; (2) elevated FSH relative to LH; (3) low ovarian and serum testosterone, and low AMH; (4) faster follicular maturation; and (5) shorter menstrual cycles. As described in detail below, all of these conditions are met by endometriosis.

The diametric model proposed here hypothesizes that risk of endometriosis is mediated in notable part by low prenatal testosterone exposure, which primes the developing HPG axis to under‐produce testosterone relative to estradiol throughout adult life, contributing to the core symptoms and correlates of endometriosis. Under the diametric disorders hypothesis, endometriosis and PCOS thus represent opposite and extreme manifestations of testosterone‐mediated HPG axis development and activity in women, with high levels increasing PCOS risk and with low levels increasing endometriosis risk. If this hypothesis is correct, then the risk factors, proximate causes, symptoms, and correlates of endometriosis and PCOS should tend to be opposite to one another. We tested this hypothesis through interdisciplinary and integrative comparisons of the two disorders, using data from the literature.

## 2 METHODS

We searched the endometriosis and PCOS literatures extensively for data on aspects of early development, endocrine‐level physiology, physical morphology, reproductive‐system physiology, life history, and epidemiology that could be compared between the two disorders. We focused especially on sets of integrated traits associated with prenatal development (including roles for testosterone) and HPG axis functioning. The findings were outlined and organized into a set of six main predictions that stem from the diametric disorders hypothesis.

## 3 RESULTS

### 3.1 Endometriosis and PCOS are associated with low and high prenatal testosterone, respectively

Sexually dimorphic morphological traits that develop under hormonal influences during specific windows of early embryological life are used as proxies for in utero testosterone exposure. Anogenital distance is a sensitive and reliable biomarker of fetal testosterone exposure across mammalian species, with elevated testosterone resulting in longer AGDs (as found in males) and reduced testosterone resulting in shorter AGDs (as found in females) (Thankamony et al., 2016). Exogenous testosterone given during early to mid‐gestation increases female AGD in diverse animal models including rodents, monkeys, and sheep (Abbott et al., 2012; DeHaan et al., 1987; Hotchkiss et al., 2007; Rhees et al., 1997). High doses of prenatal estrogenic chemicals decrease AGD in rodents, apparently through their ability to suppress testosterone production (Stewart et al., 2018). Thus, although AGD is generally used as a proxy for prenatal testosterone exposure, AGD—and reproductive development more broadly—involves complex interactions between testosterone and estrogen (Barrett et al., 2014; Williams et al., 2001).

Studies on rodents and rabbits demonstrate linear increases in female AGD as a function of in utero proximity to male siblings, with the shortest AGDs measured in females with no adjacent male siblings (0M females) and the longest AGDs in females with two adjacent male siblings (2M females) (Bánszegi et al., 2009; Clemens, 1974; Vandenbergh & Huggett, 1995; vom Saal, 1976; vom Saal et al., 1990). Testosterone concentrations in blood and amniotic fluid are higher in 2M than 0M female mouse fetuses, supporting the use of intrauterine position and AGD as metrics of prenatal testosterone exposure (vom Saal & Bronson, 1980a).

Women with PCOS have significantly longer AGDs than unaffected controls (Table 1), supporting the hypothesis that PCOS involves elevated prenatal exposure to testosterone (Filippou & Homburg, 2017). Daughters of mothers with PCOS also demonstrate evidence of longer AGDs than daughters of unaffected women (Barrett et al., 2018; Perlman et al., 2020, but see Glintborg et al., 2019), in association with high serum testosterone in affected mothers during gestation. Clinical characteristics of PCOS, including elevated serum testosterone and increased ovarian follicle count, are also positively associated with AGD in nonclinical samples of female college students (Mendiola et al., 2012; Mira‐Escolano et al., 2014).

**TABLE 1:** Comparison of developmental and morphological traits between women with endometriosis and women with polycystic ovary syndrome (PCOS)

Trait	Trait–hormone relationship	Women with endometriosis	Women with PCOS
Anogenital distance (AGD)	Reliable biomarker positively associated with early prenatal testosterone exposure (Thankamony et al., 2016)	Shorter AGD in women with endometriosis relative to unaffected women (Crestani et al., 2020; Mendiola et al., 2016; Peters et al., 2020; Sánchez‐Ferrer, Mendiola, Jiménez‐Velázquez, et al., 2017)	Longer AGD in women with PCOS relative to unaffected women (Hernández‐Peñalver et al., 2018; Peters et al., 2020; Sánchez‐Ferrer, Mendiola, Hernández‐Peñalver, et al., 2017; Sánchez‐Ferrer, Mendiola, Jiménez‐Velázquez, et al., 2017; Simsir et al., 2019; Wu et al., 2017)
Digit ratio (2D4D)	Biomarker negatively associated with prenatal testosterone exposure (Manning, 2011)	No significant difference between women with and without endometriosis (Peters et al., 2020) Higher 2D4D in women with heavy menstrual bleeding and dysmenorrhea (Tabachnik et al., 2020)	Lower 2D4D in women with PCOS relative to unaffected women in 3/5 studies, no difference in 2/5 studies (Cattrall et al., 2005; Lujan et al., 2010; Pandit et al., 2016; Peters et al., 2020; Roy et al., 2018)
Waist‐to‐hip ratio (WHR)	Low WHR predicts high mid‐cycle estradiol (Jasieńska et al., 2004), which increases conception probability (Venners et al., 2006) and cycle regularity (Singh & Singh, 2011) High WHR predicts high testosterone, and lower conception rates during in vitro fertilization (Mondragón‐Ceballos et al., 2015; Sowers et al., 2001; Van Anders & Hampson, 2005; Zaadstra et al., 1993)	Lower WHR in women with endometriosis relative to controls (Backonja et al., 2016; Byun et al., 2020)	Higher WHR in women with PCOS relative to controls (Adali et al., 2008; Cosar et al., 2008)
Body mass index (BMI)	BMI increases with testosterone levels (Mondragón‐Ceballos et al., 2015) and predicts reproductive outcomes, with reduced fecundity at BMI extremes (Gaskins et al., 2015)	Reduced BMI in affected women relative to controls (Backonja et al., 2017; Jenabi et al., 2019; Liu & Zhang, 2017), especially in severe disease, though relationship between BMI and severity is not linear (Lafay Pillet et al., 2012; Yi et al., 2009)	Above‐normal BMI and obesity in PCOS women relative to controls, independent of age, geographic region, and diagnostic criteria (Balen et al., 1995; Lim et al., 2012; Sam, 2007; Wang et al., 2018)
Fat distribution	Estrogen promotes fat deposition below waist; androgens increase abdominal fat (Dumesic et al., 2016; Motta‐Mena & Puts, 2016)	Fat distribution below the waist associated with endometriosis (Backonja et al., 2016)	Abdominal fat associated with PCOS, including lean women with PCOS (Carmina et al., 2007; Kirchengast & Huber, 2001; Lim et al., 2012)
Muscle mass	Androgens promote growth and maintenance of lean muscle mass (Notelovitz, 2002)	Lower muscle mass in affected women relative to unaffected women (Backonja et al., 2017)	Higher muscle mass in affected women relative to unaffected women (Carmina et al., 2009)

In contrast to women with PCOS, women with endometriosis demonstrate significantly shorter AGDs than unaffected women, across three datasets (Table 1). The strengths of these associations of AGD with endometriosis are substantial: In Mendiola et al. (2016), 91% of females with deep‐infiltrating endometriosis had AGDs below the median value, compared with 38% of controls (OR = 41.6, *p* = 0.002); in Crestani et al. (2020), the mean AGD of women with any degree of endometriosis as determined by laparoscopy was significantly shorter than the AGD of control subjects (OR = 6.0, *p* < 0.0001); and in Peters et al. (2020), women with deep‐infiltrating endometriosis had significantly shorter AGDs compared with controls (OR = 2.8, *p* < 0.001). Short AGD also predicts endometriosis with high specificity (91.4% in Mendiola et al., 2016; 98.6% in Crestani et al., 2020). These results strongly support the prediction that endometriosis is associated with reduced prenatal testosterone.

A second proxy for prenatal hormone exposure is the ratio of length of the second to fourth finger (2D4D) (Manning, 2011). Digit ratio reflects the ratio between testosterone and estrogen during early embryological development, with higher testosterone relative to estrogen negatively predicting 2D4D (Lutchmaya et al., 2004; Zheng & Cohn, 2011). Thus, females typically demonstrate higher 2D4D than males, and higher 2D4D predicts shorter AGD in females of humans and Rhesus macaques (Abbott et al., 2012; Barrett et al., 2015; Manning, 2011). Overall, women with PCOS exhibit lower 2D4D than controls, though not all studies report significant group differences (Table 1). The only study assessing digit ratio in women with endometriosis found that affected women had higher right‐hand 2D4D than controls, but this difference was not significant, which may be due to lack of statistical power (*n* = 43 in each group; Peters et al., 2020). Another study found that higher 2D4D predicts heavy menstrual bleeding and dysmenorrhea (Tabachnik et al., 2020), which are core symptoms of endometriosis.

### 3.2 Endometriosis and PCOS are associated with opposite hormonal profiles for hormones produced by, or interacting with, the HPG axis

We focus on a set of hormones—LH, FSH, AMH, testosterone, sex hormone‐binding globulin (SHBG), estradiol, β‐endorphins, oxytocin, kisspeptin, and activin/inhibin—that play central roles in orchestrating the female HPG axis. Table 2 summarizes the contrasting hormonal profiles between women with endometriosis and women with PCOS.

**TABLE 2:** Comparison of hormonal profiles between endometriosis and polycystic ovary syndrome (PCOS)

Hormone	Relevant functions	Activity, level in endometriosis relative to controls	Activity, level in PCOS relative to controls
Luteinizing hormone (LH)	Pituitary hormone that stimulates ovulation and corpus luteum development (Jeong & Kaiser, 2006)	Decreased, two surges	Increased, absent surge
Follicle‐stimulating hormone (FSH)	Pituitary hormone that stimulates follicular maturation and estrogen secretion (Jeong & Kaiser, 2006)	Increased	Decreased
Anti‐Müllerian hormone	Secreted by granulosa cells of large pre‐antral and small antral follicles, stimulates LH, inhibits FSH (Barbotin et al., 2019)	Decreased	Increased
Testosterone	Produced by ovarian theca cells, regulates folliculogenesis and decidualization (Couse et al., 2006; Gervásio *et al*., 2014; Gibson et al., 2016)	Decreased	Increased
Sex hormone‐binding globulin	Influences bioavailability of sex hormones (Goldštajn et al., 2016)	Increased	Decreased
Estradiol	Secreted by granulosa cells, regulates development of female sex characteristics and endometrial proliferation (Rajkovic et al., 2006)	Low‐normal (serum), High in lesions	Normal‐high (serum), No mid‐cycle peak
β‐Endorphin	Pituitary‐produced peptide that inhibits gonadotropin‐releasing hormone (GnRH) and ovulation (Plein & Rittner, 2018; Sprouse‐Blum et al., 2010)	Decreased	Increased
Oxytocin	Neurohormone released from posterior pituitary that regulates uterine peristalsis (Gimpl & Fahrenholz, 2001)	Increased	Decreased
Kisspeptin	Hypothalamic protein that initiates GnRH secretion (Skorupskaite et al., 2014)	Mixed	Increased
Activin	Ovarian cytokine that stimulates FSH, decreases LH (Seachrist & Keri, 2019)	Increased	Decreased
Inhibin	Ovarian cytokine that restrains activin (Seachrist & Keri, 2019)	Decreased	Increased

#### 3.2.1 LH and FSH

Women with PCOS demonstrate increased frequencies and amplitudes of GnRH and LH pulses, increased LH levels, reduced FSH and an increased LH to FSH ratio, and a reduced capacity to mount the LH surge that initiates ovulation (Chang & Cook‐Andersen, 2014; Chen et al., 2015; Coyle & Campbell, 2019; McCartney & Campbell, 2020). The granulosa cells from small antral follicles of women with PCOS prematurely switch from FSH to LH responsiveness, as indicated by elevated LH receptor expression and reduced FSH receptor expression compared with unaffected women (Franks & Hardy, 2020; Kanamarlapudi et al., 2016; Owens et al., 2019). These altered LH‐FSH dynamics, combined with elevated ovarian androgens, contribute to the follicular arrest, anovulation, and cyst formation characteristic of PCOS (Franks & Hardy, 2020).

Women with endometriosis demonstrate reduced LH levels, indicating a lower frequency of GnRH and LH pulses (Cahill et al., 1995; Cheesman et al., 1982; Tummon et al., 1988). Several studies report elevated FSH in women with endometriosis compared with unaffected women (de Carvalho et al., 2010; González‐Fernández et al., 2011; Romanski et al., 2019; Yoo et al., 2011), though a few studies found no differences (Cahill et al., 1995; Lima et al., 2006). Endometriosis can thus be characterized overall, in contrast to PCOS, as involving a reduced LH to FSH ratio (Li et al., 2019). The premature switch to LH responsiveness of PCOS granulosa cells also contrasts with findings from endometriosis: Women with endometriosis demonstrate lower concentrations of LH receptors in the ovarian follicles throughout the menstrual cycle (Kauppila et al., 1982; Rönnberg et al., 1984) and their cycles involve longer follicular phases marked by delayed surges of LH (Cahill et al., 1995).

Menstrual cycles of women with endometriosis more frequently demonstrate two LH surges compared with the typical single surge recorded in control subjects (90% of endometriosis cycles showed biphasic LH surges in Cheesman et al., 1982, and 17% in Vaughan Williams et al., 1986), which contrasts with the absence of an LH surge, and corresponding anovulation, found in PCOS. Taken together, this evidence shows that endometriosis and PCOS exhibit opposite deviations from unaffected women in patterns of LH and FSH production and effects.

#### 3.2.2 Anti‐Müllerian hormone

AMH regulates sex‐specific early development and mediates ovarian reserve (Barbotin et al., 2019; Lv et al., 2020). The characteristic arrest of antral follicular development in PCOS results in high AMH (Zhao et al., 2019). AMH levels are higher in anovulatory compared to ovulatory women with PCOS (Cimino et al., 2016) and also positively predict symptom severity, including hyperandrogenism and polycystic ovarian morphology (Garg & Tal, 2016; Sahmay et al., 2014).

Women with endometriosis demonstrate reduced serum AMH relative to control subjects (Dong et al., 2019; Kasapoglu et al., 2018; Muzii et al., 2018; Romanski et al., 2019; Sánchez‐Ferrer et al., 2019; Shebl et al., 2009). Women with endometriosis treated with ovarian surgery demonstrate an especially steep decline in AMH levels, but women with endometriosis who do not undergo surgery also show accelerated declines in AMH levels relative to unaffected women (Goodman et al., 2016; Kasapoglu et al., 2018; Muzii et al., 2018; Romanski et al., 2019). AMH levels also decrease with increasing severity of endometriosis (Shebl et al., 2009). Endometriosis and PCOS are thus associated with opposite levels of AMH, which also reflect diametric patterns in ovarian aging and menopause onset, as described in more detail below.

#### 3.2.3 Testosterone, sex hormone‐binding globulin, and estradiol

Elevated ovarian androgen production is a core feature of PCOS and results in PCOS symptoms including hair loss, acne, oily skin, and accumulation of abdominal fat (Rosenfield & Ehrmann, 2016). Levels of SHBG, a protein that transports steroids in biologically inactive form, are reduced in PCOS, permitting higher levels of bioavailable androgens (Deswal et al., 2018). Elevated androgen production contributes to functional changes in the adipose tissue of women with PCOS, generating chronic low‐grade inflammation across multiple tissues (Cooke et al., 2016; Fuertes‐Martín et al., 2019).

Women with PCOS demonstrate normal or elevated levels of serum estradiol relative to controls (Kawwass et al., 2017; Laven et al., 2002), but they exhibit reduced ovarian aromatase activity with a corresponding decrease in the ovarian estrogen‐to‐testosterone ratio (Chen et al., 2015; Hunter & Sterrett, 2000; Jakimiuk et al., 1998; Kirilovas et al., 2006). Low FSH and epigenetic changes in regulatory regions of the aromatase gene CYP19A1 are associated with the aromatase deficiency and androgen excess in PCOS (Franks et al., 2008).

In contrast to PCOS, endometriosis is associated with increased SHBG and reduced serum and follicular testosterone (Frankfurter et al., 1997; Misao et al., 1995; Ono et al., 2014; Panidis et al., 1993). One study found that women with endometriosis had higher serum testosterone than unaffected controls, but this difference was not statistically significant (Evsen et al., 2014). More severe disease predicted lower follicular testosterone in another study (Pellicer et al., 1998). Following a period of gonadotropin suppression prior to in vitro fertilization (IVF), women with endometriosis showed reduced follicular testosterone relative to unaffected women (Rodríguez‐Tárrega et al., 2020). Reduced testosterone, or hypoandrogenemia, is characteristic of chronic inflammatory diseases—a diverse range of conditions that involve long‐term immune activation—as testosterone generally promotes energy storage and thus inhibits metabolically costly inflammation (Straub, 2014).

Endometriosis is described as an estrogen‐dependent, chronic inflammatory disease (Bulun et al., 2019). Women with endometriosis show elevated estradiol in their menstrual blood, but not in their circulation, indicating high local estradiol production (Stilley et al., 2012; Takahashi et al., 1989). Such increased local estradiol production appears to result from reduced expression of estrogen‐metabolizing enzymes and increased aromatase expression (Attar & Bulun, 2006; Bulun et al., 2004; Hudelist et al., 2007; Huhtinen et al., 2012; Mori et al., 2019). The eutopic endometrium of women with endometriosis demonstrates significantly elevated aromatase activity compared with unaffected women (Bukulmez et al., 2008), and within affected women, aromatase expression positively predicts severity of dysmenorrhea (Maia et al., 2012).

Some studies report lower levels of aromatase expression in the ovaries and endometrium of women with endometriosis compared with controls, indicating that aromatase expression varies with ethnicity, menstrual cycle phase, fertility status, and disease severity and subtype, as well as methodology used (Anupa et al., 2019; Barcelos et al., 2015). Findings from research with swine indicate that aromatase activity is influenced by prenatal testosterone exposure: When pregnant swine were treated with flutamide, an androgen receptor antagonist, their female offspring later demonstrated increased aromatase expression and elevated ovarian estradiol production (Grzesiak et al., 2012). Overall, endometriosis can be characterized as a condition involving increased estrogenic relative to androgenic activity, whereas PCOS is characterized as the reverse.

#### 3.2.4 Endogenous opioid system

The endogenous opioid system is a sexually dimorphic modulator of the HPG axis that regulates immunity, analgesia, and stress (Böttcher et al., 2017; Eyvazzadeh et al., 2009). Females exhibit lower levels of β‐endorphin and lower pain thresholds, relative to males (Hashmi & Davis, 2014; Wiesenfeld‐Hallin, 2005). Women with PCOS demonstrated higher β‐endorphin than controls in one study (Kiałka et al., 2016). Another study found no group differences in β‐endorphin levels, but follicular β‐endorphin positively predicted serum testosterone (Jaschke et al., 2018). Prenatal testosterone excess causes adult female rats to demonstrate male‐typical responses to morphine (Cicero et al., 2002). In women with PCOS, opioids stimulate LH and insulin secretion, which increases androgen levels (Eyvazzadeh et al., 2009). Thus, hyperandrogenemia and hyperinsulinemia are worsened by an over‐active opioid system and opioid antagonists improve PCOS symptoms, as described below.

Women with endometriosis, including women with pain‐free endometriosis, demonstrate reduced β‐endorphin compared with unaffected women (Vercellini et al., 1992). Women with endometriosis symptoms also show lower pain thresholds than women without endometriosis symptoms (Poli‐Neto et al., 2019). Experimental induction of endometriosis in rats reduced opioid receptor expression by 20%, suggesting that the presence of endometriosis interferes with analgesia (Torres‐Reverón et al., 2016). Thus, in contrast to PCOS, endometriosis appears to involve an under‐active opioid system.

#### 3.2.5 Oxytocin

Oxytocin, a neurohormone with diverse effects on the brain and body, regulates uterine peristalsis (Kunz & Leyendecker, 2002). Serum oxytocin and uterine peristalsis are elevated in women with endometriosis relative to controls (He et al., 2016; Leyendecker et al., 2004). Furthermore, endometrial oxytocin receptor expression is higher in affected women and positively predicts dysmenorrhea (Harada, 2013; Huang et al., 2017). Serum oxytocin is, by contrast, reduced among women with PCOS compared to controls (Jahromi et al., 2018), and the observed frequency of uterine peristalsis is also reduced (Leonhardt et al., 2012). Type 2 diabetes and obesity, which frequently co‐occur with PCOS (Diamanti‐Kandarakis & Dunaif, 2012), are also associated with reduced serum oxytocin (Qian et al., 2014; Yuan et al., 2016).

#### 3.2.6 Kisspeptin

The peptide kisspeptin, primarily expressed in the hypothalamus, regulates GnRH secretion, sex steroid feedback, and puberty onset in both sexes (Skorupskaite et al., 2014). Serum kisspeptin is higher in patients with PCOS than in control individuals across 12 studies (Tang et al., 2019), and its concentration is positively correlated with levels of free testosterone (Chen et al., 2010; Ibrahim et al., 2020). Given the stimulating effect of kisspeptin on GnRH neurons, evidence for elevated kisspeptin in women with PCOS fits with findings of higher‐frequency GnRH and LH pulses in affected women.

One study found lower levels of kisspeptin in endometrial stroma among patients with endometriosis than in controls (Abdelkareem et al., 2020), and another study found no difference between groups (Timologou et al., 2016). To the best of our knowledge, there are no data on serum kisspeptin in women with endometriosis, but under the diametric hypothesis, lower serum kisspeptin, with corresponding reductions in GnRH and LH secretion, is expected.

#### 3.2.7 Activin and inhibin

Activin and inhibin are protein complexes with opposite biological effects: Activin contributes to menstrual cycle regulation through stimulating FSH production and decreasing testosterone, while inhibin reduces FSH synthesis and secretion (Seachrist & Keri, 2019). Women with endometriosis demonstrate higher levels of activin than unaffected controls (Cahill & Hull, 2000; Reis et al., 2012; Rombauts et al., 2006) and giving activin to mice promotes endometriotic lesion growth (Kasai et al., 2019). Conversely, women with PCOS are characterized by lower activin (Eldar‐Geva et al., 2001; Norman et al., 2001) and higher inhibin (Babćová et al., 2015; Tsigkou et al., 2008), compared with controls.

Taken together, these findings indicate that endometriosis and PCOS are characterized by opposite alterations to key hormones that regulate the female HPG axis.

### 3.3 Endometriosis and PCOS demonstrate opposite alterations to reproductive physiological processes

Given the roles of the HPG axis and its associated hormones in orchestrating reproductive functions including follicular maturation, ovulation, menstrual cycling, and uterine preparation for implantation, it follows from the diametric hypothesis that endometriosis and PCOS will involve opposite alterations in these reproductive physiological functions.

#### 3.3.1 Folliculogenesis

High ovarian testosterone promotes early follicular growth, mediating the recruitment of follicles from the primordial stage to the small, pre‐antral stage, and contributing to elevated antral follicle count, follicular arrest, anovulation, and the cyst formation characteristic of PCOS (Astapova et al., 2019). Women with PCOS thus demonstrate a slower transition from the primordial reserve to the dynamic reserve, with stalled development at the antral stage (Monniaux et al., 2014).

Lower testosterone in women with endometriosis shows evidence of contributing to a faster pace of follicular recruitment and death, across several studies. In ovaries affected by endometriosis, a higher proportion of primordial follicles are recruited into the growing pool and a greater number of maturing follicles degenerate, resulting in quicker depletion of ovarian reserve (Kitajima et al., 2014; Takeuchi et al., 2019). Women with endometriosis have fewer pre‐ovulatory follicles as well as smaller follicles, relative to unaffected women (Garcia‐Velasco & Arici, 1999; Stilley et al., 2012). Low serum testosterone predicts elevated pro‐apoptotic factors in the follicular fluid of women with endometriosis, which increase follicular atresia (Ono et al., 2014). These findings are consistent with results from swine, in which female offspring of flutamide‐treated mothers demonstrate fewer follicles, greater activation of primordial follicles, and more apoptotic cells (Knapczyk‐Stwora et al., 2013, 2019). These contrasting patterns of folliculogenesis help to account for early menopause in endometriosis and later menopause in PCOS, as discussed below.

Diametric dynamics of folliculogenesis are also observable in settings of assisted reproduction. As part of IVF, women receive doses of synthetic gonadotropins to stimulate their ovaries to rapidly mature several oocytes for harvest and fertilization (Siristatidis et al., 2012). During ovarian stimulation, women with endometriosis require higher doses of gonadotropin and produce fewer mature oocytes compared to infertile women without endometriosis (Al‐Azemi et al., 2000; Bourdon et al., 2018; González‐Fernández et al., 2011; Muteshi et al., 2018). Furthermore, correlates of endometriosis, including low LH to FSH ratios, short AGD, and early menarche, predict poor response to ovarian stimulation in women without endometriosis (Fabregues et al., 2018; Kofinas & Elias, 2014; Prasad et al., 2013; Sadrzadeh et al., 2003; Shrim et al., 2006).

In contrast to women with endometriosis, women with PCOS require lower than typical doses of gonadotropin in IVF treatment and produce more mature oocytes (González‐Fernández et al., 2011). PCOS is also associated with an increased risk of ovarian hyperstimulation syndrome, a serious condition resulting from supraphysiologic levels of gonadotropins (Kumar et al., 2011; Tummon et al., 2005), whereas endometriosis involves a decreased risk of this syndrome (Luke et al., 2010). In regularly menstruating women without endometriosis or PCOS, serum testosterone levels positively predict number of follicles and oocytes retrieved (Sun et al., 2014; Xiao et al., 2016), and women with higher serum testosterone require less FSH and a shorter duration of ovarian stimulation (Frattarelli & Peterson, 2004). Furthermore, giving testosterone to poor gonadotropin responders (who overlap with endometriosis in having low LH to FSH and short AGDs) increases retrieved oocytes and improves IVF success (Bosdou et al., 2012; Luo et al., 2014; Noventa et al., 2019; but not in Sunkara et al., 2011).

These findings indicate that women with PCOS and women with endometriosis demonstrate opposite responses to controlled ovarian stimulation, which can be understood in the context of diametric patterns of ovarian androgens and folliculogenesis.

#### 3.3.2 Menstrual cycles

Women with PCOS demonstrate lengthened menstrual cycles, due to anovulation, absent luteal progesterone, and interrupted negative feedback on gonadotropin secretion (Franks & Hardy, 2020). Women with endometriosis, by contrast, demonstrate shorter menstrual cycles relative to unaffected women (Arumugam & Lim, 1997; Wei et al., 2016; Yasui et al., 2015). Endometriosis also involves an apparent elevated incidence of releasing two eggs due to biphasic LH surges, as described above. Female rodents prenatally exposed to low testosterone also demonstrate shorter estrus cycles, as well as higher rates of conception (Table 3).

**TABLE 3:** Comparison of reproductive traits between female rodents differing in intrauterine testosterone exposure (0M females exposed to lower testosterone; 2M females exposed to higher testosterone), and women with endometriosis compared to polycystic ovary syndrome (PCOS)

Trait	Rodents	References	Women	References
Anogenital distance	Lower in 0M females than in 2M females	Clemens et al. (1978), Keisler et al. (1991), vom Saal and Bronson (1978), vom Saal et al. (1990), Zehr et al. (2001)	Lower in endometriosis females than in controls Higher in PCOS females than in controls	see Table 1 references
Timing, onset of estrus, menarche	Earlier in 0M females than in 2M females	Clark and Galef (1998), vom Saal (1981, 1989)	Earlier in endometriosis females than in controls Later in PCOS females of normal BMI than in controls	Day et al. (2015), Nnoaham et al. (2012) Carroll et al. (2012), Sadrzadeh et al. (2003), Welt and Carmina (2013)
Estrus, menstrual cycle duration	Shorter in 0M females than in 2M females	Clark and Galef (1998), vom Saal (1981, 1989), vom Saal and Bronson (1980b)	Shorter in endometriosis females than in controls Longer, irregular in PCOS females than in controls	Arumugam and Lim (1997), Wei et al. (2016), Yasui et al. (2015) Franks and Hardy (2020)
Fertility and fecundity measures	Higher in 0M females than in 2M females	Clark and Galef (1998), Drickamer (1996), Drickamer et al. (2001)	Reduced in endometriosis and PCOS (as diseases) 0M female twins have higher fecundity than 1M female twins Endometriosis associated with traits linked to higher fertility (low waist‐to‐hip ratio, BMI)	Bulun et al. (2019), Rosenfield and Ehrmann (2016) Bütikofer et al. (2019), Lummaa et al. (2007) see Table 1 references

#### 3.3.3 Decidualization

Decidualization, the collective biochemical and morphological changes to the endometrium that prepare it for implantation (Okada et al., 2018), is dysregulated in both endometriosis and PCOS. Aspects of decidualization in endometriosis can be characterized as overexpressed. Women with endometriosis demonstrate more mobile endometrial stromal cells, higher numbers of uterine natural killer cells, reduced regulation over endometrial invasion, as well as up‐regulated angiogenesis, pro‐inflammatory cytokine expression, and oxidative stress (Brosens et al., 2012; Gellersen & Brosens, 2014; Lessey et al., 2013; Matteo et al., 2017; Sharpe‐Timms, 2001; Soares et al., 2012; Xavier et al., 2005). Indeed, Somigliana et al. (1999) hypothesized that immunological alterations to the endometria and other tissues of women with endometriosis promote excellent endometrial receptivity, in the absence of severe effects from endometriosis such as adhesions and anatomical obstructions. Furthermore, these alterations to decidualization contribute directly to the establishment and growth of ectopic endometrial tissue, the primary hallmark of this disorder (Patel et al., 2017).

Women with PCOS, in contrast to those with endometriosis, demonstrate incomplete and delayed decidualization (Younas et al., 2019). Genes involved in adhesion, invasion, and tissue remodeling are downregulated in endometrium of women with PCOS, causing reduced trophoblast invasion which contributes to increased rates of preeclampsia and early pregnancy loss (Brosens & Benagiano, 2015; Piltonen et al., 2015). Women with PCOS also demonstrate reduced numbers of uterine natural killer cells (Matteo et al., 2010). Treatment with metformin, a drug prescribed for diabetes as well as PCOS, improves markers of endometrial receptivity in women with PCOS, including increased uterine blood flow (Palomba et al., 2006). Recent research with a hyperandrogenic mouse model showed that angiogenesis and uterine natural killer cells are increased with flutamide treatment, demonstrating that elevated testosterone levels inhibit decidualization (Gong et al., 2019).

To summarize, women with endometriosis demonstrate shorter menstrual cycles with preserved or even increased ovulatory rates, faster testosterone‐mediated depletion of ovarian reserve, poor response to exogenous ovulatory stimulation, and exaggerated aspects of decidualization. Women with PCOS demonstrate longer menstrual cycles with infrequent or absent ovulation, slower testosterone‐mediated depletion of ovarian reserve, strong response to ovarian stimulation, and reduced aspects of decidualization. These opposite sets of alterations from typical HPG functioning and associated hormonal activity contribute directly to the symptoms and fertility reductions of both conditions.

### 3.4 Endometriosis and PCOS are associated with opposite morphological traits

Testosterone and estradiol play important roles in shaping sexually dimorphic characteristics including body size and shape (Mondragón‐Ceballos et al., 2015). In this context, PCOS is associated with morphological traits indicating elevated testosterone, and lower estrogen‐to‐testosterone ratios, including higher body mass index (BMI) and higher waist‐to‐hip ratio (WHR), which are driven by increased abdominal adiposity and higher muscle mass (Table 1).

In contrast to PCOS, women with endometriosis demonstrate physical attributes associated with lower testosterone, higher estrogen, or both, including lower BMI, lower WHR, reduced abdominal fat, lower muscle mass, and a gynoid pattern of fat distribution on the hips and buttocks (Table 1).

In humans, lower WHR and lower BMI have been associated, across multiple studies including cross‐cultural analysis, with measures of higher female “attractiveness” to males, and with higher fecundity or reproductive value (Andrews et al., 2017; Cloud & Perilloux, 2014; Weeden & Sabini, 2005). Among mice, rabbits, and gerbils, 0M females (that developed under lower prenatal testosterone) demonstrate higher attractiveness to males than 2M females, across multiple studies employing a range of mate choice paradigms (Clark & Galef, 1998; Rines & vom Saal, 1984; vom Saal & Bronson, 1978, 1980a). For example, odors of female rabbits with shorter AGDs elicit a stronger mating response from males, and these females produce larger, heavier litters (Bánszegi et al., 2012). These findings suggest that, among humans as well as nonhuman mammals (Table 3), females exposed to low testosterone in early development demonstrate phenotypic attributes that signal high fertility and reproductive value.

### 3.5 Endometriosis and PCOS are associated with opposite life‐history traits

The HPG axis proximately instantiates life‐history decision‐making, integrating bodily information with environmental cues to coordinate the development and expression of key life‐history events such as onset of puberty and reproductive senescence (Segner et al., 2017).

Women with endometriosis show slightly but significantly earlier ages at menarche relative to unaffected women (≤12 years; Table 3). Women with PCOS demonstrate the opposite pattern: Young women of normal weight who later develop PCOS are more likely to have had delayed or absent menarche (≥16 years; Table 3). Women with PCOS who reported themselves as overweight during menarche onset were more likely to report younger age at menarche (Welt & Carmina, 2013). This pattern is consistent with results from animal research: 0M females mature, mate, and conceive at earlier ages than 2M females (Table 3). Earlier‐maturing female gerbils are also more fecund, birthing more litters with more young per litter (Clark & Galef, 1998).

Reproductive senescence also shows opposite patterns between the two conditions. Women with endometriosis reach menopause at significantly earlier ages than unaffected women (Pokoradi et al., 2011; Yasui et al., 2011), whereas women with PCOS reach menopause significantly later than unaffected women (Forslund et al., 2019; Minooee et al., 2018; Tehrani et al., 2010).

The two conditions also demonstrate diametric associations with multiple proxies of ovarian reserve. Compared to unaffected women, women with PCOS demonstrate elevated numbers of immature follicles, and their pool of potential oocytes declines more slowly through time, as reflected by a slower reduction in AMH (Hudecova et al., 2009; Mulders et al., 2004; Nikolaou & Gilling‐Smith, 2004). Endometriosis, by contrast, is associated with lower ovarian reserve (Seyhan et al., 2015; Shah, 2013), increased primordial follicle recruitment (Takeuchi et al., 2019), fewer antral follicles (Muzii et al., 2018), and a steeper rate of decline in AMH (Kasapoglu et al., 2018).

The effects of low versus high prenatal testosterone exposure on several phenotypes, including life‐history traits, can usefully be compared between human women and nonhuman female mammals. Table 3 shows that low versus high prenatal testosterone exposure has consistent effects on female development and reproduction between rodents and humans.

**FIGURE 1:**
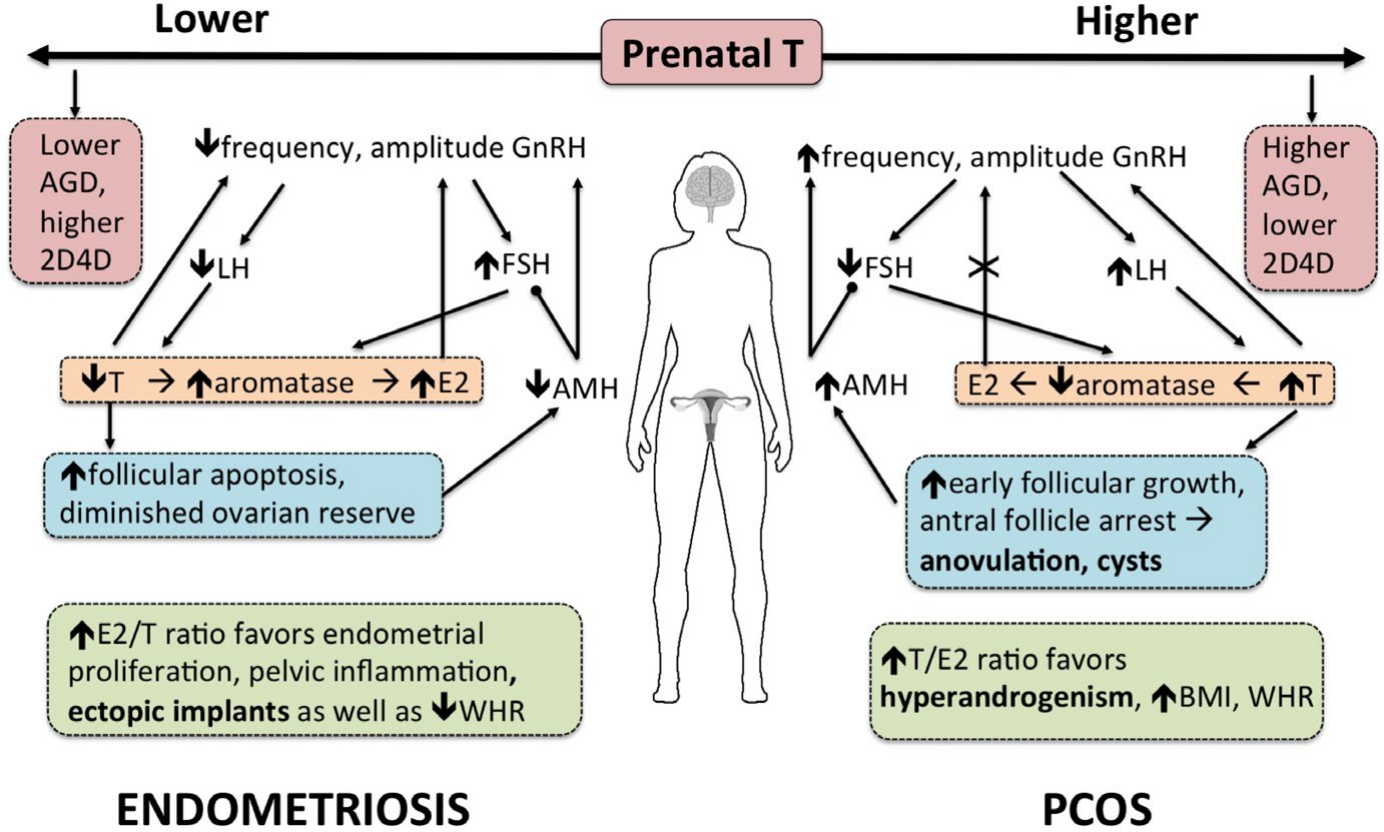
The diametric disorder hypothesis for endometriosis and polycystic ovary syndrome (PCOS) proposes that opposite levels of prenatal testosterone exposure (low in endometriosis; high in PCOS) program the developing hypothalamic–pituitary–gonadal axis, resulting in under‐production (in endometriosis) and over‐production (in PCOS) of ovarian testosterone relative to estradiol in adulthood. Pointed arrowheads indicate a stimulating effect (e.g., GnRH stimulates LH) and rounded arrowheads indicate an inhibitory effect (e.g., AMH inhibits FSH). The positive feedback effect of E2 on GnRH release is interrupted in PCOS, denoted by X. Altered testosterone to estradiol ratios in both conditions directly contribute to their symptoms (noted in bold). 2D4D, second to fourth finger ratio; AGD, anogenital distance; AMH, anti‐Müllerian hormone; BMI, body mass index; E2, estradiol; FSH, follicle‐stimulating hormone; GnRH, gonadotropin‐releasing hormone; LH, luteinizing hormone; T, testosterone; WHR, waist‐to‐hip ratio

Figure 1 summarizes the diametric phenotypes of endometriosis and PCOS, in the context of the diverse lines of evidence described above.

### 3.6 Endometriosis and PCOS rarely occur together

If endometriosis and PCOS are diametric disorders, then they should tend to not co‐occur within individuals; thus, the diametric hypothesis predicts that rates of endometriosis should be lower or nonexistent in women with PCOS. This prediction needs to be evaluated with careful consideration of comparison groups, as even among women with no self‐reported symptoms of endometriosis (pain, heavy bleeding, infertility), laparoscopic examination conducted for other reasons sometimes reveals evidence of endometriosis. To evaluate this prediction, we draw on data that compares rates of discovered endometriosis between women with PCOS and women free of PCOS and endometriosis symptoms.

Moghadami‐Tabrizi et al. (1998) compared PCOS patients to asymptomatic women (women undergoing tubal ligation who had no symptoms of PCOS or endometriosis) and found that the asymptomatic women had a significantly higher prevalence of endometriosis lesions (19.9%) than did the women with PCOS (7.3%); for both groups, the endometriosis was reported as minimal or mild. A meta‐analysis of studies assessing the presence of endometriosis in women with PCOS undergoing ovarian drilling found that, overall, 7.7% of women with PCOS demonstrated evidence of endometriosis, all of which was minimal or mild (Hager et al., 2019).

These rates of about 7–8% of asymptomatic endometriosis phenotypes in women with PCOS can be compared with results from studies that looked for evidence of asymptomatic endometriosis among women who were undergoing tubal ligation. These studies yielded rates of endometriosis that center around 19% (Barbosa et al., 2009; Rawson, 1991; Tissot et al., 2017). Holoch et al. (2014) reported high rates of endometriosis in women with PCOS (>70%), but the PCOS group was subject to ascertainment bias because the women were examined for endometriosis only if they self‐reported pelvic pain and/or infertility. Taken together, these studies suggest that, among women without any clear symptoms of endometriosis (pain, heavy bleeding, infertility), the rate of this disorder as determined by laparoscopy is about one‐half to one‐third lower in women with PCOS than in women with no known reproductive issues.

## 4 DISCUSSION

**FIGURE 2:**
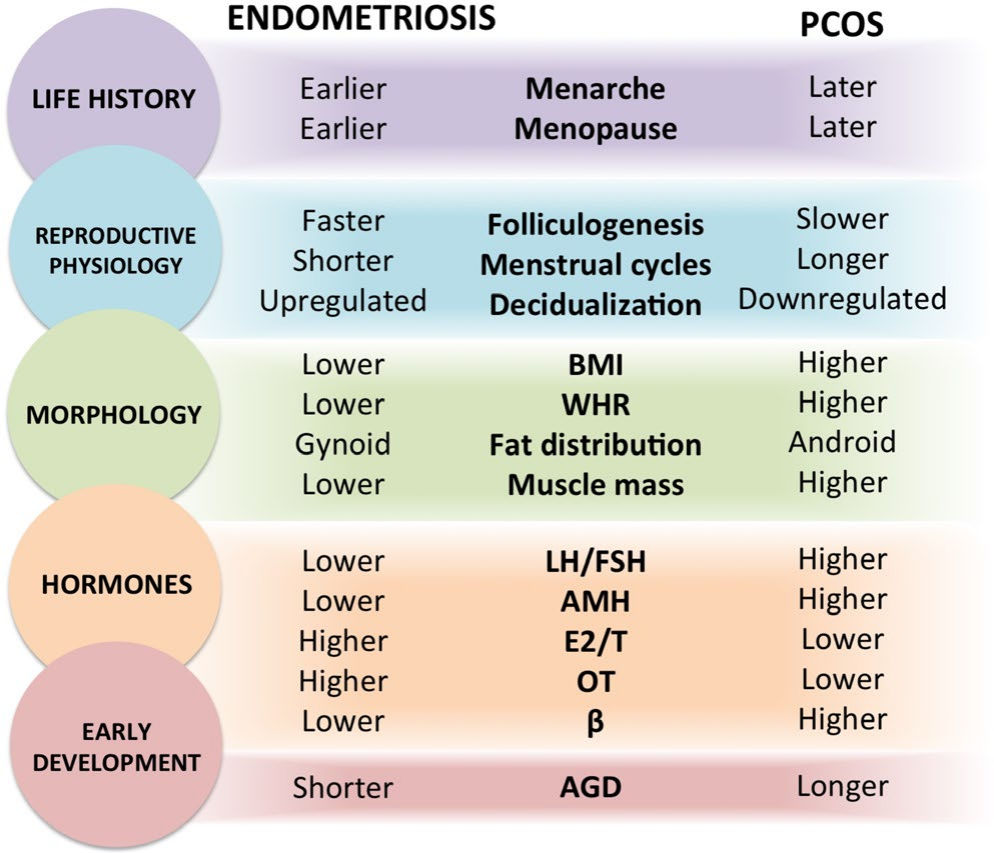
Diametric phenotypes between endometriosis and polycystic ovary syndrome (PCOS) are evident across multiple categories, including early testosterone‐mediated development (red), adult endocrinological activity (orange), body morphology (green), reproductive physiology (blue), and life history (purple). AGD, anogenital distance; AMH, anti‐Müllerian hormone; BMI, body mass index; E2/T, estradiol relative to testosterone; LH/FSH, luteinizing hormone relative to follicle‐stimulating hormone; OT, oxytocin; WHR, waist‐to‐hip ratio; β, β‐endorphin

We have evaluated the hypothesis that endometriosis and PCOS represent opposite, extreme, and dysregulated manifestations of variation in female HPG axis development and functioning, with risk strongly modulated by levels of prenatal testosterone. The developmental and endocrine bases for the hypothesis are summarized in Figure 2. Whereas elevated prenatal testosterone is a well‐established cause of PCOS (Abbott et al., 2019; Filippou & Homburg, 2017), understanding endometriosis as a developmental disorder involving reduced prenatal testosterone exposure is a novel contribution with diverse clinical, research, and theoretical implications. Our review of evidence supports the hypothesis that endometriosis and PCOS represent diametric disorders that reflect extreme expressions of evolutionary–ecological trade‐offs (Crespi & Go, 2005), proximately mediated by variation in prenatal development and HPG functioning across the female reproductive lifespan.

Multiple lines of evidence support the hypothesis that, in contrast to PCOS, endometriosis involves low prenatal testosterone: (1) Women with endometriosis demonstrate significantly shorter AGDs than unaffected women; (2) female rodents naturally exposed to low prenatal testosterone demonstrate short AGDs, as well as earlier reproductive maturation and shorter estrus cycles, which are correlates and risk factors of endometriosis; (3) endometriosis symptoms (heavy, painful menstruation) are associated with reduced prenatal testosterone, as indexed by digit ratio; (4) endometriosis involves a set of HPG alterations (low LH to FSH, low AMH, low ovarian T) that are opposite to the HPG alterations characteristic of PCOS that, from a large body of animal research, are known to be caused by prenatal testosterone excess; and (5) blocking androgen activity during fetal development increases ovarian estradiol production in swine, and elevated ovarian estradiol is a major proximate cause of endometriosis. These independent lines of evidence converge in revealing a causal role for low prenatal testosterone in the development of endometriosis.

Diverse evidence also indicates that low prenatal testosterone primes the adult HPG axis to under‐produce ovarian testosterone and that low adult testosterone contributes to endometriosis symptoms and correlates: (1) Women with endometriosis demonstrate reduced levels of follicular and serum testosterone; (2) AGD is positively correlated with circulating testosterone in healthy women; (3) low serum testosterone in women with endometriosis increases the rate of follicular recruitment and atresia, contributing to diminished ovarian reserve and earlier menopause of affected women; (4) testosterone supplementation increases ovarian response to exogenous hormones; (5) chronic inflammatory diseases, a category to which endometriosis belongs, are characterized by hypoandrogenemia; and (6) women with endometriosis tend to demonstrate a suite of morphological phenotypes that centrally involve reduced testosterone relative to estradiol, including low BMI and WHR. Together, these lines of evidence support the diametric model's framing of core endometriosis symptoms and correlate as involving low ovarian and circulating testosterone, with developmental roots in low prenatal testosterone.

That *high* prenatal testosterone programs the adult female HPG axis to continually over‐produce ovarian testosterone is well documented in humans and several animal models. How *low* prenatal testosterone impacts female HPG development and activity, and subsequent reproduction, has received significantly less research attention, perhaps due to conceptual biases in the literature toward higher, but not lower, testosterone being considered as physiologically and developmentally causal.

Accounts of endometriosis have largely focused on the role of estrogen, as estrogen proximately contributes to lesion growth through its proliferative and inflammatory effects on endometrial tissue. Such characterization of endometriosis as an estrogen‐dependent disease (Bulun et al., 2019) may have inadvertently overshadowed the role of testosterone in endometriosis etiology. For example, recent replicated findings of short AGD in women with endometriosis (Crestani et al., 2020; Mendiola et al., 2016; Peters et al., 2020) were, in each article, interpreted as indicative of an overly estrogenic prenatal environment. Estrogenic prenatal environments do decrease AGD (Stewart et al., 2018) and increase endometriosis risk (Missmer et al., 2004), so this interpretation is accurate but incomplete. Given that AGD is generally used as a proxy for prenatal testosterone exposure and that AGD predicts adult testosterone levels but not estrogen (Eisenberg et al., 2012; Mira‐Escolano et al., 2014), it is clearly important to address both estrogen and testosterone when investigating endometriosis etiology.

Positioning this expanded awareness of how estrogen and testosterone together mediate risk of female reproductive disorders in an evolutionary context provides productive avenues for further research. Given that estrogen and testosterone represent key female and male sex hormones, endometriosis and PCOS can be conceptualized as existing at opposite ends of a spectrum of variation in sexually dimorphic traits, within females (Dinsdale et al., 2021). Traits typically observed in females relative to males, including short AGD, gynoid physique, low β‐endorphin and testosterone, and elevated oxytocin, are thus especially highly expressed in women with endometriosis. In an evolutionary medical context, this suite of phenotypes may be framed as representing an extreme expression of female reproductive adaptations. Because prenatal testosterone plays a crucial role in mediating sex differentiation in utero, endometriosis may be fruitfully explored as involving an especially female‐biased trajectory of reproductive development.

A second evolutionary context through which to understand the diametric relationship between endometriosis and PCOS is life‐history trade‐offs. Framing endometriosis and PCOS as opposite and extreme expressions of life‐history strategies is supported by the clear continuity of prenatal testosterone effects on reproductive life histories across females of multiple mammalian species (Table 3). Correlates of endometriosis, particularly earlier menarche and menopause and rapid menstrual cycling, indicate a life‐history strategy where early investment into reproduction is favored. In both traditional and modern populations, younger age at menarche predicts earlier first pregnancy as well as higher fecundability (Guldbrandsen et al., 2014; Hochberg et al., 2011). High investment into early reproduction is expected to entail survival costs, which should be greater in conditions of poor resource availability. Indeed, Lycett et al. (2000) reported a negative relationship between fecundity and longevity for women with low access to resources. Another study found that a faster pace of reproduction with higher parity resulted in higher mortality and poorer nutritional condition, though only in the short term (Gurven et al., 2016).

Polycystic ovary syndrome, involving later menarche and menopause and extended or absent menstrual cycling, may represent a maladaptive extreme of a strategy where investment into maintenance and survival is favored over reproductive effort, particularly in challenging environments (reviewed in Corbett & Morin‐Papunen, 2013). Investment into visceral fat—which is elevated in lean as well as obese women with PCOS—appears to enhance health and survival in harsh conditions of low nutrition and high infection (West‐Eberhard, 2019; Lassek & Gaulin, 2018). Studies of mice indicate that, in environments with high population densities, females prenatally exposed to high testosterone may be more effective competitors for limited resources (vom Saal & Bronson, 1980a). Higher prenatal testosterone exposure could thus be beneficial for females in environments where resource competition is high and traits such as increased robustness, muscularity, and dominance provide survival and competitive benefits. Contrasting PCOS and endometriosis as extreme expressions of life‐history strategies provides insights into the costs and benefits of prenatal testosterone exposure to female development and behavior, an area that has received little attention thus far.

A central strength of the diametric hypothesis is that it is capable of unifying previous hypotheses for the causes of endometriosis. One widely accepted explanation for endometriosis is that, in some women, refluxed endometrial cells from menstruation are insufficiently cleared from the peritoneal cavity, and then implant and proliferate into lesions (Sampson, 1927). The diametric hypothesis addresses the puzzle of why, although 90% of women experience retrograde menstruation (Halme et al., 1984), only 5%–10% of women develop endometriotic lesions: (1) Low prenatal and postnatal testosterone contributes directly and indirectly to more menstruations and greater menstrual blood volume via early menarche, shorter menstrual cycles, and thicker endometrial lining; (2) high local estradiol relative to testosterone increases inflammatory responses to ectopic tissue in women with endometriosis; and (3) elevated oxytocinergic activity increases uterine contractility, increasing the likelihood and volume of reflux menstruation.

Another important hypothesis for the etiology of endometriosis is based on inflammatory activation of developmentally mislocated stem cells, or Müllerian fragments (Russell, 1899; Sasson & Taylor, 2008). By the diametric model, such events are mediated by effects of prenatal testosterone on expression of reproductive developmental genes including HOXA10 (Cermik et al., 2003; He et al., 2018) as well as other genes involved in cell apoptosis, migration, and proliferation (Knapczyk‐Stwora et al., 2019; Laganà et al., 2017).

The diametric disorder hypothesis for endometriosis and PCOS is subject to important caveats and limitations. There are research areas where existing evidence is limited or absent. For example, connections of AGD with testosterone and follicular count come from one dataset (e.g., Murcia Young Women's Study; Mendiola et al., 2012; Mira‐Escolano et al., 2014) and thus require replication. More studies examining testosterone levels and correlations between testosterone levels and endometriosis symptoms and severity are also needed.

Evidence of endometriosis lesions in women with PCOS indicates that causes of endometriosis apart from prenatal testosterone exposure should also be considered. This co‐occurrence is low, and such overlap might be explained in the following three ways. Firstly, endometriosis can be induced by treatments for PCOS (and vice versa) (Dinsdale et al., 2021), which could account for some of the co‐occurrence. Secondly, when endometriosis is discovered in women with PCOS, it is mild. Risk factors for endometriosis that are strongly linked to low prenatal testosterone, such as short AGD, are especially predictive of severe endometriosis. Mild endometriosis may be framed as a possible outcome of normal inflammation involved in female reproductive processes. An analogy to cancer can be applied here, whereby some people and families are especially prone to specific, aggressive kinds of cancer, but anyone can develop cancerous growths under certain environmental or lifestyle conditions. Environmental exposures (e.g., endocrine‐disrupting chemicals) can also dysregulate normal endocrine and inflammatory processes and may reduce the threshold for endometriosis lesions in women who, under the diametric model, would be considered low risk for the disease. Thirdly, aspects of PCOS, such as high testosterone, could independently contribute to endometriosis lesions through increasing susceptibility to infection, which is a hypothesized proximate pathway whereby endometriosis lesions begin (García‐Peñarrubia et al., 2020).

There are other examples of phenotypic similarity between PCOS and endometriosis, such as inflammation and progesterone resistance, but these appear to manifest differently and involve different causes (Li et al., 2014; Patel et al., 2017). Under the diametric hypothesis, areas of phenotypic overlap are expected to involve opposite proximate causes in the two conditions, but further study is required. A final limitation to consider is that, to the best of our knowledge, the effects of prenatal androgen deficiency have only been experimentally assessed in swine, whereas the effects of prenatal androgen excess on the developing female HPG axis are well characterized from experiments on diverse animal models. Future studies that experimentally analyze the proximate basis of endometriosis in prenatal development, coupled with work that focuses on the adaptive evolutionary trade‐offs and extremes involved in risks of endometriosis and PCOS, should provide substantial new insights into the causes and treatments of both disorders.

## CONFLICT OF INTEREST

The authors declare no conflict of interest.

## Data Availability

Data sharing is not applicable to this article as no new data were created or analyzed in this study.
